# Impact on Mental Well-Being and Resilience of Patients with Multiple Chronic Conditions in Different Periods during the Coronavirus Disease 2019 Outbreak in Taiwan

**DOI:** 10.3390/healthcare9111457

**Published:** 2021-10-27

**Authors:** Yu-Yin Kao, Yi-Chun Chen, Tsuen-Wei Hsu, Hua-Rong Zhong, Ben-Chung Cheng, Chien-Te Lee, Chen-Hsiang Lee

**Affiliations:** 1 Department of Nursing, Kaohsiung Chang Gung Memorial Hospital, College of Nursing, Kaohsiung Medical University, Kaohsiung 80708, Taiwan; yuyin0020@cgmh.org.tw; 2 Department of Internal Medicine, Division of Infectious Diseases, Kaohsiung Chang Gung Memorial Hospital, College of Medicine, Chang Gung University, Kaohsiung 83301, Taiwan; sonice83@cgmh.org.tw; 3 Department of Internal Medicine, Division of Nephrology, Kaohsiung Chang Gung Memorial Hospital, College of Medicine, Chang Gung University, Kaohsiung 83301, Taiwan; scwei@cgmh.org.tw (T.-W.H.); gvboy@cgmh.org.tw (H.-R.Z.); benz@cgmh.org.tw (B.-C.C.); ctlee33@cgmh.org.tw (C.-T.L.)

**Keywords:** coronavirus disease 2019, mental well-being, resilience, chronic condition

## Abstract

Concerns over the coronavirus disease 2019 (COVID-19) pandemic and control measures have affected the routine outpatient visits of individuals with comorbidities and their mental well-being. From October 2019 to August 2020, this cross-sectional study enrolled 135 patients who sought medical attention at a medical center in Taiwan. This period covered the early (October to December 2019), peak (January to April 2020), and late (May to August 2020) periods of the COVID-19 outbreak in Taiwan. The demographic data, social support data, activities of daily living (ADL), resilience scale scores, and mental well-being scale scores of the participants were compared. There were no statistically significant differences in the participation rate, demographic data, and social support data between the three periods. The correlation analysis confirmed significant negative relationships between the number of COVID-19 cases and outpatient department visits per month (r = −0.764, *p* < 0.001), emergency department visits per month (r = −0.023, *p* < 0.001), ADL (r = −0.257, *p* = 0.03), resilience scale (r = −0.390, *p* < 0.001), and mental well-being scale (r = −0.475, *p* < 0.001). In conclusion, the severity of the COVID-19 outbreak in Taiwan was associated with declines in the ADL, mental well-being, and resilience of patients who sought medical attention.

## 1. Introduction

Coronavirus disease 2019 (COVID-19) has spread to 187 countries, with >3 million reported deaths worldwide [1]. Reports from China and the United States have suggested that older patients with multiple chronic conditions are at a higher risk of developing severe COVID-19 outcomes than the general population [2]. In addition, patients with COVID-19 and chronic conditions, such as diabetes, hypertension, heart disease, and chronic lung disease, were also associated with high mortality rates [3,4].

Taiwan was affected by the COVID-19 pandemic in early 2020. Through the experience with the severe acute respiratory syndrome (SARS) epidemic in Taiwan in 2003 and the early implementation of strict control measures by the National Health Command Center when COVID-19 was first reported in China, the incidence of confirmed COVID-19 cases was significantly lower in Taiwan than in most other countries [5]. Nevertheless, anxiety over the COVID-19 pandemic caused an extensive decline in the number of routine outpatient visits [6]. This reduction in routine outpatient visits and loss of patients with comorbidities during follow-up may have affected the management and control of their chronic diseases [7].

Weakness is considered to be a pre-debilitating stage by the World Health Organization, and the onset of frailty was associated with a 50% increase in the risk of mortality [8]. Elderly patients with multiple chronic diseases are more likely to lose their ability to respond to acute problems, which, in turn, is more likely to lead to acute functional decline and changes in cognitive function compared with younger patients [9]. Castellana et al. reported that, compared to patients with a robust physical status, physically frail patients had a hazard ratio of 1.78 (95% confidence interval: 1.25–3.19, *p* < 0.01) for death [10]. Integrating the complex medical needs of elderly patients can help to increase resilience and prevent the progression of debilitating conditions. Resilience during hospitalization is also an important issue, especially in the recovery process, regarding adapting and adjusting to the feeling of the self-control of limb function [11]. Some long-term studies on pandemics were conducted in Asian countries during the SARS pandemic [12]. According to these studies, quarantined individuals had a high prevalence of psychological distress and disorders [13,14]. Several psychosocial factors, including sex, occupation, environment, and self-esteem, were also associated with the level of resilience [15,16]. Mental well-being is an important psychosocial outcome [17,18]. However, the impact of the COVID-19 pandemic on the mental well-being and resilience of patients with multiple chronic diseases during different periods of the pandemic has rarely been investigated. Therefore, the purpose of this study was to analyze the demographic, economic, and mental health correlates of different periods during the COVID-19 outbreak in Taiwan. We aimed to identify factors that could predict improvements or exacerbations of psychological distress.

## 2. Materials and Methods

### 2.1. Study Design and Setting

This was a prospective observational study. From October 2019 to August 2020, consecutive patients without COVID-19 who were admitted via the emergency department to an integrated medical ward at a 2700-bed tertiary care hospital in southern Taiwan were screened for eligibility to participate in this cross-sectional survey. The inclusion criteria were patients aged 20–90 years who were able to read and comprehend questionnaires and were willing to complete the questionnaire interview. If the patients had more than one episode of admission, only the first episode was included. The enrolled participants were classified into three groups according to the time of admission and the COVID-19 situation in Taiwan from October 2019 to August 2020 as follows: early COVID-19 period (October to December 2019), peak COVID-19 period (January to April 2020), and late COVID-19 period (May to August 2020). On average, there were 176,398 and 11,304 visits per month at the outpatient and emergency departments, respectively, at this institution in 2019 before the COVID-19 pandemic. During the study period, there were no changes in the indications for hospitalization or screening criteria for eligibility of the participants.

The demographic characteristics, Charlson comorbidity index, acute illness at admission, income, activities of daily living, resilience scale scores, and mental well-being scale scores were evaluated during the patients’ hospitalization. Activities of daily living, resilience scale scores, and mental well-being scale scores were also evaluated before discharge. All participants provided written informed consent. This study was approved by the Institutional Review Board (IRB) of Kaohsiung Chang Gung Memorial Hospital (IRB number: 202000417B0C501).

### 2.2. Data Collection Methods

The number of hospital outpatient/emergency visits was measured using data from the hospital. Data on demographic characteristics, including sex, age, marital status, educational level, employment status, religion, and Charlson comorbidity index [19] were collected. The Charlson comorbidity index is a validated, simple, and readily applicable method of estimating the risk of death from comorbid diseases and it has been widely used as a predictor of long-term prognosis and survival [19]. International Classification of Diseases, Tenth Revision (ICD-10) codes were used to record the diseases of the patients at admission [20]. Household income before tax was classified by asking the participant to indicate the category that best represented the total personal income of all family members (including the patient) in the past 12 months. The first and fourth quartiles represented the lowest and highest income levels, respectively. In addition, activities of daily living were assessed using the Barthel index score as follows: 0–20 (totally dependent), 21–60 (severe dependence), 61–90 (moderate dependence), 91–99 (slight dependence), and 100 (independent) [21]. Resilience and mental well-being scores were also recorded. The resilience scale [22] consists of 25 questions covering the following five domains: (1) meaning of life, (2) calm mind areas, (3) retention of confidence area, (4) indomitable spirit areas, and (5) acceptance of the existence of solitary areas. The total score ranges from 25 to 175 points, with a higher score indicating better resilience. The mental well-being scale was developed by Dupuy [23] and subsequently translated into Chinese [24]; it was used to measure the well-being of the respondents. The scale consists of 18 items, including six dimensions: anxiety, depression, general health, positive mental well-being, self-control, and vitality. The total score ranges from 0 to 110 points, with a higher score indicating better mental well-being. There are three levels of distress according to the total score: 0–60 (severe distress), 61–72 (moderate distress), and 73–110 (positive well-being).

### 2.3. Statistical Analysis

Sample size calculations were performed using G*Power 3.1.9.2 software (Franz, Universitat Kiel, Germany). All data were analyzed using SPSS software version 20.0 (IBM Corp., Armonk, NY, USA). Descriptive statistics were calculated to examine the distribution of the study variables among the participants. Categorical variables were analyzed using an independent sample t-test and analysis of variance of the three periods of the COVID-19 outbreak in Taiwan. Spearman correlation coefficient analysis was used to examine correlations between the number of COVID-19 cases, Charlson comorbidity index, outpatient department visits per month, emergency department visits per month, activities of daily living, resilience scale scores, and mental well-being scale scores. Statistical significance was set at *p* < 0.05.

## 3. Results

### 3.1. Participant Characteristics of the Different Periods of the COVID-19 Outbreak in Taiwan

During the 10-month study period, 2,066 patients were admitted to the integrated medical ward. Among them, 799 patients who met the inclusion criteria were invited to participate in the study, of whom, 135 returned the questionnaires without any missing data. The minimum sample size was calculated as 90 after setting the effect size at 0.3 (minimum size), alpha error at 0.05, and power at 0.80. Therefore, the sample size of this study (n = 135) had sufficient statistical power to detect differences. The participation rates were 17.1, 16.9, and 17.0% in the early, peak, and late periods of the COVID-19 outbreak in Taiwan, respectively (Table 1). Among the enrolled patients, 87 were male and 48 were female, including 45 (31 males and 14 females) with a mean age of 61.27 ± 16.37 years in the early period, 38 (22 males and 16 females) with a mean age of 64.74 ± 17.48 years in the peak period, and 52 (34 males and 18 females) with a mean age of 58.75 ± 19.33 years in the late period. There were no statistically significant differences in the participation rate, sex, age, marital status, education level, employment status, religion, income quartiles, Charlson comorbidity index, and classification of acute diseases at admission between the participants enrolled during the three periods. However, there were significant differences in outpatient department visits per month (*p* < 0.001), emergency department visits per month (*p* < 0.001), activities of daily living (*p* = 0.015), resilience scale scores (*p* < 0.001), and mental well-being scale scores (*p* < 0.001) between the three periods (Table 2).

### 3.2. Variable Correlations

**Figure 1:**
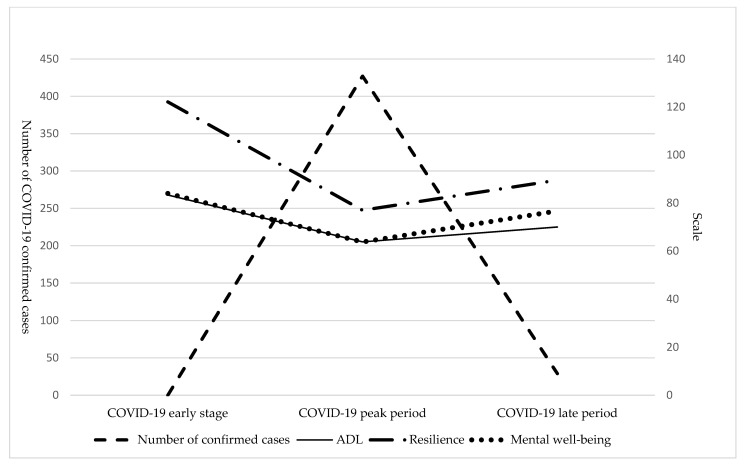
Number of confirmed COVID-19 cases in Taiwan and activities of daily living, mental well-being, and resilience for the different outbreak periods. At the peak of the outbreak, the activities of daily living, mental well-being, and resilience were significantly lower than during the early and late periods. Correlation analysis confirmed significant negative relationships between the number of COVID-19 cases and the resilience scale (r = −0.378, *p* < 0.001), mental well-being scale (r = −0.438, *p* < 0.001), and activities of daily living (r = −0.177, *p* = 0.04). ADL, activities of daily living.

Correlation analysis (Table 3) confirmed significant negative relationships between the number of COVID-19 cases and the outpatient department visits per month, activities of daily living, resilience scale, and mental well-being scale. This reflected that at the peak of the outbreak, the activities of daily living, mental well-being, and resilience of the patients were significantly affected and were lower than during the early and late periods of the outbreak (Figure 1). The results also revealed significant negative relationships between the Charlson comorbidity index and the activities of daily living, resilience scale, and mental well-being scale. In addition, there were significant positive relationships between the outpatient department visits per month and the activities of daily living, resilience scale, and mental well-being scale. Furthermore, there were significant positive relationships between the emergency department visits per month and the activities of daily living, resilience scale, and mental well-being scale.

### 3.3. Activities of Daily Living at Admission and Discharge during the COVID-19 Outbreak in Taiwan

**Figure 2:**
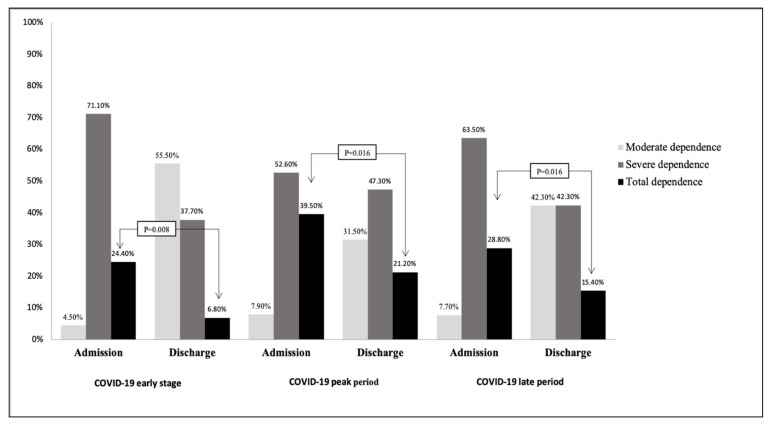
Activities of daily living at admission and discharge during the COVID-19 outbreak in Taiwan. Among the three periods, the number of patients who were totally dependent at admission was highest during the peak period and similarly at discharge. Compared to the number of patients who were totally dependent at admission, fewer patients were totally dependent at discharge, regardless of the period of the COVID-19 outbreak in Taiwan.

Among the three periods, the percentage (39.5%) of patients who were totally dependent at admission was highest during the peak period of the COVID-19 outbreak in Taiwan compared to the early stage (24.4%) and late period (28.8%). Compared with the number of patients who were totally dependent at admission, fewer patients were totally dependent at discharge, regardless of the period of the COVID-19 outbreak (all, *p* < 0.05, Figure 2). However, the percentage (21.2%) of patients who were totally dependent at discharge was still the highest during the COVID-19 peak period compared to the early stage (6.8%) and late period (15.4%). This showed that the activities of daily living of the patients were correlated with the different stages of the COVID-19 outbreak in Taiwan.

## 4. Discussion

To provide adequate mental health interventions during the COVID-19 pandemic, it is necessary to understand the impact of the COVID-19 pandemic on mental well-being and resilience. In this study, we explored the effect of the COVID-19 outbreak on the activities of daily living, mental well-being, and resilience during the peak period of the outbreak compared to the early and late periods in Taiwan. We found that the peak period of the COVID-19 outbreak was associated with significant reductions in outpatient department and emergency department visits, even though there was no community outbreak in Taiwan. Although there were no significant differences in the classification of acute illnesses, comorbidities, and social support during the three periods, we found a significant reduction in the activities of daily living at admission during the peak period (Table 1). This finding is consistent with observations from other developed countries [25,26,27,28] and may have been due to anxiety over the pandemic, resulting in delays in accessing health care for chronic illnesses that require regular medical follow-ups. Moreover, this was reflected by the significant reductions in outpatient and emergency department visits and delays in admissions, which may have increased the severity and complexity of diseases that require medical attention and timely management [29]. This may have been correlated with the reduction in the mental well-being and resilience of patients at admission [30,31]. After the COVID-19 outbreak had been controlled in Taiwan (the late period), patients were more willing to visit the outpatient department and to be admitted to the hospital via the emergency department, just as in the early period of the COVID-19 outbreak (Table 1).

The Charlson comorbidity index score was negatively associated with the activities of daily living, resilience, and mental well-being. Our results confirmed that the perceived threat to health by the pandemic led to uncertainty and fear, increased stress, and vulnerability, which subsequently had a detrimental impact on subjective mental well-being. During the COVID-19 pandemic, most countries have adopted confinement measures to reduce in-person exposure. Several studies reported that patients were not able to recover or achieve the expected outcomes, leading to disappointment or helplessness and poor adaptation, resulting in negative emotions and behavior [32,33,34]. As a result, COVID-19 public health restrictions influence those who are physically unwell and weak and are significantly correlated with enthusiasm for disease treatment and damage to mental health [35]. Resilience is less likely to recover during the COVID-19 pandemic, and mortality has significantly increased [36]. Older patients with multiple chronic conditions are more likely to have debilitating conditions and reductions in activities of daily living and mobility, leading to a limited range of activities and reduced self-care ability. A recent study reported that hospitalization for respiratory distress due to SARS-CoV-2 infection was associated with a relatively long recovery period and poor prognosis [37]. The Taiwanese government implemented various preventive measures during the COVID-19 outbreak, including public health education, medical resource allocation, mandatory use of facemasks, enhanced case identification, high-risk group quarantine, and restrictions on the number of visitors to patients in healthcare facilities and hospitals [38]. However, the number of non-emergency hospitalizations and elective surgery admissions significantly decreased, and the medical conditions of inpatients during the same period were generally more severe and required medical care [38]. Compared with the patients who were totally dependent at admission, fewer patients were totally dependent at discharge, regardless of the period of the COVID-19 outbreak (Figure 2).

The numbers of patients with depressive and anxiety disorders globally were estimated to have increased by about 50 million and 80 million, respectively, in 2020 as a result of the COVID-19 pandemic [39]. These increases have both been associated with increasing SARS-CoV-2 infection rates and decreasing human mobility. Mitigation strategies could incorporate ways to promote mental well-being, target determinants of poor mental health, and provide interventions to treat those with mental disorders [39]. Governments should promote clear communication strategies because social media and news outlets may provide confusing information and thereby increase fear and anxiety. Communication campaigns could promote messages encouraging preventive actions to avoid the spread of the virus [40]. Digital platforms may serve as alternatives to promote social support and contact with family and friends, which are elements that promote resilience [41]. In addition, telemedicine, including video or phone consultations, can allow individuals with chronic conditions to self-monitor symptoms and send this information to their clinicians via mobile apps and/or other digital platforms [42]. Telehealth during the pandemic is increasingly important, and the innovative adoption of digital technologies can continue to provide valuable patient–clinician communication, not only for clinical care but also for in-person primary care, specialty care, and monitoring behavioral changes in patients [43,44].

There are several limitations to this study, including the extremely low levels of documented COVID-19 infections in Taiwan compared with those in most other countries during the study period. Furthermore, the sample size was relatively small; therefore, we did not analyze subcategories of resilience and well-being in this study. However, the sample size (n = 135) of this study had sufficient statistical power to detect differences after a sample size power estimation, and there were no statistically significant differences in the participation rate and demographics between the participants who were enrolled during the three periods. In addition, the study was performed over 10 months during the COVID-19 pandemic; therefore, conclusions about the long-term effects cannot be inferred. Moreover, the cross-sectional study design meant that we could not determine the directionality of the observed relationships. Experimental studies should address the directionality of these effects. In addition, we used data from the outpatient and emergency departments of a tertiary hospital; thus, our findings have limited external validity. Despite the reduction in the number of outpatient department and emergency department visits, we could not determine whether the patients visited other healthcare facilities. Future longitudinal studies in other settings with more COVID-19 cases in the community are warranted to evaluate changes in mental health due to the evolution of the pandemic.

## 5. Conclusions

The topic explored in this study is important because the mental health of patients with chronic debilitating diseases is associated with poor quality of life indicators and contributes significantly to the overall burden of disease. We found that the increase in anxiety during the COVID-19 peak period was associated with a significant reduction in outpatient department and emergency department visits, leading to negative psychological consequences. The reduction in healthcare system utilization during the peak of the outbreak resulted in decreased mental well-being, resilience, and activities of daily living. These results highlight the importance of these psychosocial factors in shaping community responses to pandemics.

## Figures and Tables

**Table 1:** Participant characteristics of the different periods of the COVID-19 outbreak in Taiwan.

Variable	COVID-19 Early Stage	COVID-19 Peak Period	COVID-19 Late Period	*F*	*p*
	*n* (%)	*n* (%)	*n* (%)		
Number of COVID-19 cases	0	428	29		
n/N	45/263 (17.1)	38/224 (16.9)	52/312 (17.0)	0.822	0.442
Age, mean ± SD, years	61.27 ± 16.37	64.74 ± 17.48	58.75 ± 19.33	1.233	0.295
Visits per month					
OPD	184,294	147,457	168,312	4.530	<0.001
ED	9861	8888	9482	1.246	<0.001
Sex				0.552	0.577
Male	31 (68.9)	22 (57.9)	34 (65.4)		
Female	14 (31.1)	16 (42.1)	18 (34.6)		
Marital status				1.961	0.145
Single	7 (15.6)	4 (10.5)	12 (23.1)		
Married/cohabitating	37 (82.2)	30 (78.9)	37 (71.2)		
Divorced/widowed	1 (2.2)	4 (7.9)	3 (5.7)		
Education level				0.837	0.435
Above college	7 (15.6)	5 (13.2)	7 (13.5)		
Senior or junior high school	20 (44.4)	15 (39.5)	30 (57.7)		
Below elementary school	18 (40.0)	18 (47.4)	15 (28.8)		
Employed				0.252	0.777
No	28 (62.2)	26 (68.4)	32 (61.5)		
Yes	17 (37.8)	12 (31.6)	20 (38.5)		
Religion				0.777	0.462
Yes	30 (66.7)	30 (78.9)	38 (73.1)		
No	15 (33.3)	8 (21.1)	14 (26.9)		
Acute diseases at admission				0.892	0.412
Certain infectious diseases	19 (42.2)	21 (55.3)	26 (50.0)		
Diseases of the circulatory system	3 (6.7)	3 (7.9)	3 (5.8)		
Diseases of the digestive system	13 (28.9)	5 (13.2)	15 (28.8)		
Diseases of the genitourinary system	1 (2.2)	4 (10.5)	4 (7.7)		
Diseases of the nervous system	9 (20.0)	5 (13.1)	4 (7.7)		
Income quartiles				1.248	0.290
1 (Poorest)	8 (17.8)	3 (7.9)	4 (7.7)		
2 (Poorer)	11 (24.4)	10 (26.3)	14 (26.9)		
3 (Middle)	23 (51.1)	19 (50.0)	29 (55.8)		
4 (Wealthiest)	3 (6.7)	6 (15.8)	5 (9.6)		

ADL, activities of daily living; CCI, Charlson comorbidity index; n, enrolled patients; N, screened patients; OPD, outpatient department; ED, emergency department; SD, standard deviation.

**Table 2:** Scale scores of the participants of the different periods of the COVID-19 outbreak in Taiwan.

Variable	COVID-19 Early Stage	COVID-19 Peak Period	COVID-19 Late Period	*F*	*p*
	Mean ± SD	Mean ± SD	Mean ± SD		
CCI	4.13 ± 1.79	4.53 ± 2.05	3.69 ± 2.08	1.977	0.143
ADL	83.33 ± 26.37	63.82 ± 35.27	70.00 ± 32.30	4.304	0.015
Resilience scale	122.11 ± 21.14	76.97 ± 20.46	89.52 ± 35.48	30.912	<0.001
Mental well-being scale	83.98 ± 12.32	63.76 ± 11.37	76.71 ± 18.93	18.961	<0.001

ADL, activities of daily living; CCI, Charlson comorbidity index.

**Table 3:** Variable correlations.

Variable		COVID-19	CCI	OPD	ED	ADL	Resilience Scale	Mental Well-Being Scale
COVID-19	*r*	1	0.176	−0.764	−0.023	−0.257	−0.390	−0.475
	*p*		0.041	<0.001	0.788	0.003	<0.001	<0.001
CCI	*r*		1	−0.080	0.094	−0.535	−0.240	−0.257
	*p*			0.358	0.278	<0.001	0.005	0.003
OPD	*r*			1	0.455	0.268	0.553	0.439
	*p*				<0.001	0.002	<0.001	<0.001
ED	*r*				1	0.209	0.346	0.172
	*p*					0.015	<0.001	<0.001

COVID-19, COVID-19 cases; CCI, Charlson comorbidity index; OPD, outpatient department visits per month; ED, emergency department visits per month; ADL, activities of daily living; *r*, Spearman correlation.

## Data Availability

The data associated with the paper are not publicly available but are available from the corresponding author on reasonable request.
